# Immune checkpoint inhibitor-associated arrhythmias: clinical challenges, artificial intelligence-enhanced monitoring, and management strategies

**DOI:** 10.3389/fcvm.2026.1834378

**Published:** 2026-09-14

**Authors:** Yi Xia, Haoyu Dong, Xiangzhu Meng, Yingxue Dong, Xiaolei Yang, Yunlong Xia

**Affiliations:** Department of Cardiology, Institute of Cardiovascular Diseases, The First Affiliated Hospital of Dalian Medical University, Dalian, China

**Keywords:** arrhythmias, artificial intelligence, cardio-oncology, cardiovascular immune-related adverse events, immune checkpoint inhibitors

## Abstract

Immune checkpoint inhibitors (ICIs) have revolutionized the therapeutic landscape for a broad spectrum of malignancies, substantially prolonging patient survival. However, the widespread clinical adoption of ICIs is challenged by a diverse array of cardiovascular immune-related adverse events (CV-irAEs). Among these, arrhythmias pose a considerable threat to patient safety and treatment continuity, given their insidious onset and potential lethality. Traditionally, these arrhythmias have been largely conceptualized as secondary complications of ICI-induced myocarditis, a perspective that has obscured their true clinical features. Emerging evidence indicates that ICI-associated arrhythmias exhibit distinct risk profiles and pathogenic mechanisms, suggesting that arrhythmias may be substantially more common than previously recognized and frequently manifest as standalone clinical entities rather than mere secondary complications. Accordingly, to mitigate cardiovascular risk and optimize long-term patient outcomes, this review synthesizes the epidemiology, risk factors, pathogenesis, and management strategies of ICI-associated arrhythmias, while highlighting the transformative potential of artificial intelligence-enhanced monitoring in advancing precision cardio-oncology.

## 1 Introduction

Immune checkpoint inhibitors (ICIs) represent a landmark breakthrough in oncology. By fundamentally reshaping the therapeutic landscape, ICIs have yielded unprecedented survival benefits across a diverse spectrum of malignancies. Consequently, these agents are integral to the standard of care for various solid tumors and hematological malignancies, including melanoma, non-small cell lung cancer, hepatocellular carcinoma, renal cell carcinoma, Hodgkin lymphoma, head and neck cancer, and urothelial carcinoma (1). Mechanistically, ICIs are monoclonal antibodies that block T-cell inhibitory pathways, thereby reversing immunosuppression, activating the immune system, and enhancing antitumor responses (2). These agents comprise two main classes: (i) Programmed Cell Death Protein 1 (PD-1) inhibitors (pembrolizumab, nivolumab, cemiplimab, toripalimab, dostarlimab, tislelizumab, and retifanlimab) and Programmed Cell Death Ligand 1 (PD-L1) inhibitors (atezolizumab, avelumab, and durvalumab); (ii) Cytotoxic T-Lymphocyte-Associated Protein 4 (CTLA-4) inhibitors (ipilimumab and tremelimumab) (3).

Despite the remarkable clinical efficacy of ICIs, their potent immune-activating properties also pose a unique set of challenges, specifically immune-related adverse events (irAEs). Among these, cardiovascular immune-related adverse events (CV-irAEs) have garnered significant attention due to their potentially fatal nature; these complications primarily manifest as arrhythmias, myocarditis, pericarditis, heart failure, acute coronary syndrome, vasculitis, and Takotsubo cardiomyopathy (4, 5). Early studies predominantly focused on myocarditis and regarded arrhythmias merely as its secondary complications (6, 7). However, a burgeoning body of evidence suggests that arrhythmias have emerged as the most prevalent CV-irAEs, frequently occurring even in the absence of myocarditis (8–10). ICI-associated arrhythmias are characterized by an insidious onset and rapid progression, rendering them highly susceptible to being overlooked by clinicians, which can lead to fatal outcomes. Therefore, a thorough understanding of the full clinical spectrum of these arrhythmias is essential to heighten clinical vigilance and ensure the cardiovascular safety of immunotherapy. This review synthesizes the current evidence on ICI-associated arrhythmias, moving beyond the conventional paradigm that regards them merely as secondary complications of myocarditis to provide a comprehensive overview of their epidemiology, risk factors, pathophysiological mechanisms, and management strategies. Given that ICI-associated arrhythmias are characterized by an insidious onset, dynamic fluctuations, and high susceptibility to missing paroxysmal episodes via standard 12-lead electrocardiography (ECG), traditional screening approaches frequently lead to under-detection. This clinical diagnostic gap drives the urgent medical need for advanced monitoring paradigms. Consequently, we bridge this clinical challenge by highlighting the forward-looking, exploratory potential of artificial intelligence (AI)-enhanced screening and smart wearables to advance precision risk-stratification.

## 2 Review methodology

To ensure a balanced and comprehensive synthesis of the literature, a structured literature search was conducted across the PubMed and Embase databases from inception through January 2026. The search strategy utilized combinations of Medical Subject Headings (MeSH) terms and free-text keywords, including “Immune Checkpoint Inhibitors,” “Antineoplastic Agents, Immunological,” “Arrhythmias,” “Atrial Fibrillation,” “Conduction Blocks,” and “Artificial Intelligence”. Only peer-reviewed articles published in English were considered.

Studies were selected based on their relevance to arrhythmias occurring during or after ICI therapy. Prospective cohorts, large-scale retrospective database analyses, pharmacovigilance studies, and mechanistic translational studies were prioritized. Conflicting evidence, particularly regarding incidence rates and agent-specific risks, was interpreted in light of study design, sample size, baseline comorbidities, and monitoring intensity.

This article is a structured narrative review rather than a systematic review or meta-analysis. No preregistered protocol was developed, and no formal risk-of-bias assessment or certainty-of-evidence grading was performed. Therefore, potential selection and interpretive biases cannot be excluded.

Because the available evidence spans heterogeneous study designs, findings were interpreted according to the inferential scope and methodological limitations of each design. Prospective cohorts using standardized serial ECGs, continuous telemetry, or ambulatory ECG monitoring may more completely detect asymptomatic and paroxysmal arrhythmias, but available cohorts are generally small and observational. Retrospective studies based on electronic health records, administrative claims, or registries provide larger sample sizes but remain susceptible to selection bias, coding errors, residual confounding, and nonuniform monitoring intensity. Spontaneous pharmacovigilance databases, including the U.S. Food and Drug Administration Adverse Event Reporting System (FAERS), can identify reporting disproportionality signals but cannot estimate true incidence, absolute or relative clinical risk, or establish causality. Case reports, case series, and mechanistic studies were used to characterize rare clinical phenotypes and generate biological hypotheses; they were not treated as confirmatory clinical evidence.

## 3 Epidemiology

ICI-associated arrhythmias encompass a broad clinical spectrum, categorized into tachyarrhythmias (e.g., atrial fibrillation, supraventricular tachycardia, and ventricular arrhythmias) and bradyarrhythmias (e.g., conduction blocks) (11). Among these, atrial fibrillation represents the most prevalent subtype. Conversely, despite their lower incidence, ventricular arrhythmias and conduction blocks typically denote more severe myocardial injury and portend a heightened risk of mortality, particularly sudden cardiac death (12). It is clinically imperative to distinguish immune-mediated electrical disturbances from secondary physiological fluctuations in heart rate, which are ubiquitous within the oncological patient population. Sinus tachycardia and sinus bradycardia are frequently driven by systemic cancer-related stressors rather than direct autoimmune injury to the cardiac conduction system. Therefore, sinus tachycardia and sinus bradycardia are excluded from the primary classification of ICI-associated arrhythmias in this review.

The overall incidence of ICI-associated arrhythmias remains a matter of ongoing debate, with reported rates spanning a wide range from 1.0% to 25.0% across multiple studies (8–10, 13–38). The characteristics of these studies are summarized in Table 1. This substantial variability is likely attributable to heterogeneity in study designs, diagnostic criteria, ICI regimens, and monitoring modalities. Notably, prospective cohorts employing rigorous serial ECG monitoring report a substantially higher incidence of arrhythmias than retrospective analyses. Prospective studies by Mirabel et al. and Haj-Yehia et al. demonstrated incidence rates of 18.4% and 25.0%, respectively (10, 17). In contrast, some retrospective cohorts relying on diagnostic codes or voluntary symptomatic presentations report rates ranging from 1.0% to 6.6%. This stark discrepancy suggests that without proactive and systematic ECG surveillance, a significant proportion of paroxysmal or asymptomatic arrhythmias remains undetected, thereby leading to a substantial underestimation of the true clinical burden. Emerging evidence indicates that new-onset arrhythmias occur more frequently in male patients and are disproportionately prevalent among elderly individuals (aged ≥65 years) (39, 40). The observed sex disparity may stem from the higher incidence of primary ICI indications (e.g., lung cancer, melanoma, and genitourinary malignancies) among men, leading to an overrepresentation of male participants in clinical trials (41–43). Additionally, a subset of female patients is often excluded from such trials due to pre-existing autoimmune conditions (e.g., rheumatoid arthritis and systemic lupus erythematosus) that contraindicate ICI therapy, potentially resulting in an underestimation of the actual arrhythmia risk in women. Thus, well-designed prospective studies are warranted to verify the validity of these sex-based differences. Age is established as a key risk factor for cancer, with disease incidence rising markedly with advancing years (44). Beyond the increased cancer burden, elderly patients frequently present with multiple comorbidities that dysregulate immune system homeostasis, thereby predisposing them to an increased risk of arrhythmic events (45). Due to limited clinical recognition and suboptimal surveillance strategies, early studies predominantly attributed arrhythmias to myocarditis, characterizing the former as secondary complications rather than direct toxic effects of ICI therapy itself (46). However, ICI-associated arrhythmias have emerged as a prominent manifestation of cardiotoxicity, occurring either in isolation or concomitantly with myocarditis (47). Indeed, mounting evidence suggests that arrhythmias have surpassed myocarditis as the most prevalent CV-irAEs (9, 10, 15, 17–19, 24–28, 30, 31, 33, 34, 37, 48–51). A real-world study utilizing the FAERS database demonstrated a steady annual increase in ICI-associated arrhythmias from 2017 to 2020. Among 1,957 confirmed cases, a substantial 77.77% presented as isolated events, while the remaining cases were accompanied by other cardiotoxicities, including myocarditis (9.66%), heart failure (9.10%), coronary artery disease (5.06%), pericarditis (3.17%), and valvular heart disease (0.46%) (52). Escudier et al. analyzed 30 patients with ICI-associated cardiotoxicity and reported that atrial fibrillation, ventricular arrhythmias, and conduction blocks occurred in 30%, 27%, and 17% of cases, respectively. Crucially, these three arrhythmic subtypes can occur in isolation in the absence of left ventricular systolic dysfunction (12). In an international multicenter retrospective study by Nassar et al., 40 out of 110 solid tumor patients treated with ICIs experienced CV-irAEs, with arrhythmias (*n* = 14) identified as the most common presentation. Notably, no cases of myocarditis or pericarditis were documented in this cohort (9). In the first prospective study evaluating the incidence of ICI-associated cardiotoxicity, 49 solid tumor patients were followed up for 6 months, during which 9 patients (18.4%) developed arrhythmias, whereas no cases of myocarditis, pericarditis, or acute coronary syndrome were detected (10).

**Table 1:** Summary of clinical studies on ICI-associated arrhythmias.

Authors/year	Study design	Patients (*N*)	Cancer type	ICI class	New-onset arrhythmia, *n* (%)	Arrhythmia endpoints	Concomitant myocarditis, pericarditis, and/or thyroiditis	Ref
Wang et al. 2025	Retrospective	9,541	Various	PD-1i, PD-L1i, CTLA-4i + PD-1i, CTLA-4i + PD-L1i	1,498 (15.7%)	All arrhythmias	Not reported	(8)
Nassar et al. 2025	Retrospective	110	Solid tumors	PD-1i, PD-L1i, CTLA-4i, CTLA-4i + PD-1i	14 (12.7%)	All arrhythmias	No myocarditis or pericarditis	(9)
Wang et al. 2025	Retrospective	203	Various	PD-1i, PD-L1i	2 (1.0%)	All arrhythmias	No myocarditis or pericarditis	(13)
Perelman et al. 2025	Retrospective	455	Various	PD-1i, PD-L1i, CTLA-4i	30 (6.6%)	Atrial fibrillation, atrial flutter, ventricular arrhythmias	Not reported	(14)
Slieman et al. 2025	Retrospective	240	Various	PD-1i, PD-L1i	15 (6.3%)	Atrial fibrillation, atrial flutter, ventricular arrhythmias	Not reported	(15)
Kwan et al. 2024	Retrospective	2,165	Various	PD-1i, PD-L1i, CTLA-4i, CTLA-4i + PD-1i, CTLA-4i + PD-L1i	444 (20.5%)	All arrhythmias	Not reported	(16)
Mirabel et al. 2024	Prospective	49	Solid tumors	PD-1i, PD-L1i, CTLA-4i + PD-1i	9 (18.4%)	Atrial fibrillation, conduction blocks, ventricular arrhythmias	No myocarditis or pericarditis	(10)
Haj-Yehia et al. 2024	Prospective	88	Various	PD-1i, PD-L1i, CTLA-4i, CTLA-4i + PD-1i	22 (25.0%)	QTc prolongation, atrial fibrillation, sinus tachycardia	Not reported	(17)
Reyes-Gibby et al. 2024	Retrospective	453	Head and neck cancer	PD-1i	109 (24.1%)	All arrhythmias	Not reported	(18)
Rong et al. 2024	Retrospective	8,175	Lung cancer	PD-1i, PD-L1i	914 (11.2%)	All arrhythmias	Not reported	(19)
Perelman et al. 2024	Retrospective	119	Various	PD-1i, PD-L1i, CTLA-4i + PD-1i	7 (5.9%)	All arrhythmias	Not reported	(20)
Son et al. 2023	Retrospective	194	Lung cancer	PD-1i, PD-L1i	9 (4.6%)	Supraventricular tachycardia, atrial fibrillation	Not reported	(21)
Ho et al. 2023	Retrospective	315	Various	PD-1i, PD-L1i, CTLA-4i	3 (1.0%)	All arrhythmias	Not reported	(22)
Andres et al. 2022	Retrospective	2,647	Various	PD-1i, PD-L1i, CTLA-4i, CTLA-4i + PD-1i	30 (1.1%)	All arrhythmias	3 cases with myocarditis, 2 cases with thyroiditis	(23)
Brumberger et al. 2022	Retrospective	538	Various	PD-1i, PD-L1i, CTLA-4i	14 (2.6%)	Atrial fibrillation, ventricular arrhythmias	Not reported	(24)
Chiang et al. 2022	Retrospective	868	Various	PD-1i, PD-L1i, CTLA-4i	27 (3.1%)	All arrhythmias	Not reported	(25)
Li et al. 2022	Retrospective	5,518	Various	PD-1i, PD-L1i, CTLA-4i, CTLA-4i + PD-1i	511 (9.3%)	All arrhythmias	Not reported	(26)
Torrente et al. 2022	Retrospective	378	Various	PD-1i, PD-L1i, CTLA-4i, CTLA-4i + PD-1i	46 (12.2%)	QTc prolongation, conduction blocks, atrial fibrillation	Not reported	(27)
Wu et al. 2022	Retrospective	495	Various	PD-1i, PD-L1i	34 (6.9%)	All arrhythmias	Not reported	(28)
Zhou et al. 2022	Retrospective	1,959	Not reported	PD-1i, PD-L1i	60 (3.1%)	Atrial fibrillation, atrial flutter	Not reported	(29)
Zhang et al. 2022	Retrospective	155	Not reported	PD-1i	8 (5.2%)	All arrhythmias	2 cases with myocarditis	(30)
Jain et al. 2021	Retrospective	12,187	Various	PD-1i, PD-L1i, CTLA-4i, CTLA-4i + PD-1i	434 (3.6%)	Atrial fibrillation, conduction blocks	Not reported	(31)
D'Souza et al. 2021	Retrospective	1,100	Lung cancer, melanoma	PD-1i, CTLA-4i	42 (3.8%)	All arrhythmias	Not reported	(32)
Waheed et al. 2021	Retrospective	424	Various	PD-1i, PD-L1i, CTLA-4i, CTLA-4i + PD-1i, CTLA-4i + PD-L1i	26 (6.1%)	All arrhythmias	Not reported	(33)
Heilbroner et al. 2021	Retrospective	4,960	Lung cancer, melanoma, renal cell carcinoma	PD-1i, PD-L1i	218 (4.4%)	All arrhythmias	Not reported	(34)
Schiffer et al. 2021	Retrospective	76	Various	Not reported	15 (19.7%)	All arrhythmias	1 cases with myocarditis	(35)
Moey et al. 2020	Retrospective	196	Lung cancer	PD-1i, PD-L1i, CTLA-4i + PD-1i	7 (3.6%)	Supraventricular tachycardia	Not reported	(36)
Cathcart-Rake et al. 2020	Retrospective	3,164	Lung cancer	PD-1i, PD-L1i	287 (9.1%)	All arrhythmias	Not reported	(37)
Chitturi et al. 2019	Retrospective	135	Lung cancer	PD-1i, PD-L1i, CTLA-4i, CTLA-4i + PD-1i	25 (18.5%)	All arrhythmias	Not reported	(38)

ICI, immune checkpoint inhibitor; PD-1i, programmed cell death protein 1 inhibitor; PD-L1i, programmed cell death ligand 1 inhibitor; CTLA-4i, cytotoxic T-lymphocyte-associated protein 4 inhibitor; QTc, corrected QT interval.

Temporally, ICI-associated arrhythmias follow a distinct pattern; while the cumulative risk correlates with the duration of therapy, events predominantly cluster in the early treatment phase (53). Notably, tachyarrhythmias typically manifest earlier than conduction blocks (54). A real-world analysis of the FAERS database reported a median onset time of 32 days for ICI-associated arrhythmias, with 48.9% of events occurring within the first 30 days (55). These findings identify the first month of ICI therapy as a critical high-risk window, underscoring the necessity for vigilant early screening and proactive management. Wang et al. investigated temporal variations in arrhythmia onset across different ICI regimens, observing that the median time to onset did not differ significantly among various monotherapies (*P* = 0.061). Specifically, the median onset times were 25 days for pembrolizumab, 35 for nivolumab, 56 for cemiplimab, 35 for durvalumab, 34 for atezolizumab, 18 for avelumab, and 47 for ipilimumab. Furthermore, no significant disparity in median onset time was observed between monotherapy and combination ICI regimens (52). Contrasting with these findings, a study involving 610 patients with head and neck cancer reported a significantly longer median onset of 4.9 months (18). This discrepancy may be attributed to the study's reliance on voluntary emergency department presentations for diagnosis. Given that many arrhythmias manifest with insidious or non-specific symptoms, patients may not seek timely medical intervention, potentially leading to an underestimation of early-onset events and an overall delay in diagnosis.

## 4 Risk factors

Risk factors for ICI-associated arrhythmias encompass both patient-specific and treatment-related factors (Table 2). A comprehensive baseline assessment is essential to facilitate risk stratification, identify high-risk individuals, and inform the development of personalized monitoring strategies and therapeutic regimens.

**Table 2:** Risk factors for ICI-associated arrhythmias.

Risk classification	Specific risk factor	Statistical association strength (95% CI)	Clinical implication & biomarker relevance	Authors/year	Ref
Cancer types	Lung cancer	HR: 1.40 (1.09–1.80); *P* = 0.007	High baseline pulmonary inflammatory environment upregulates risk.	Kwan et al. 2024	(16)
	Advanced metastatic stage	OR: 3.48 (1.36–8.87); *P* = 0.009	Enhanced tumor burden correlates with systemic immune dysregulation.	Chiang et al. 2022	(25)
Pre-existing cardiovascular diseases	Hypertension	OR: 1.75 (1.02–2.98); *P* = 0.04	Impaired baseline cardiac reserve limits tolerance to autoimmune stress.	Chiang et al. 2022	(25)
Lifestyle and inflammatory factors	Elevated baseline NLR	HR: 1.74 (1.16–2.61); *P* = 0.007	High NLR indicates subclinical hyper-inflammation.	Haj-Yehia et al. 2024	(17)
	Smoking	IRR: 4.21 (1.29–13.76); *P* = 0.017	Alters tumor microenvironment and recruits baseline T-cell infiltration.	Brumberger et al. 2022	(24)
	Elevated BMI	HR: 1.05 (1.01–1.10); *P* = 0.033	Obesity induces chronic low-grade inflammation and upregulates baseline PD-1 expression.	Reyes-Gibby et al. 2024	(18)
Combination therapy	CTLA-4i + PD-1i	ROR: 3.91 (3.01–5.07) compared to monotherapy	Dual immune checkpoint blockade induces fulminant, early-onset electrical toxicity.	Balzano et al. 2025	(59)

ICI, immune checkpoint inhibitor; PD-1i, programmed cell death protein 1 inhibitor; CTLA-4i, cytotoxic T-lymphocyte-associated protein 4 inhibitor; NLR, neutrophil-to-lymphocyte ratio; BMI, body mass index; HR, hazard ratios; OR, odds ratios; ROR, reporting odds ratios; IRR, incidence rate ratios.

### 4.1 Patient-specific risk factors

#### 4.1.1 Cancer types

Liu et al. analyzed 1,945 patients with ICI-associated arrhythmias and found that these events were most prevalent in lung cancer (32.08%), followed by melanoma (20.46%) and genitourinary malignancies (16.25%) (55). Kwan et al. demonstrated that lung cancer (HR 1.40; 95% CI: 1.09–1.80; *P* = 0.007) and genitourinary malignancies (HR 1.29; 95% CI: 1.04–1.61; *P* = 0.02) are independent risk factors for the development of ICI-associated arrhythmias (16). Data from 5,518 cancer patients treated with ICIs, extracted from a large healthcare organization network, revealed that lung cancer significantly increased the risk of arrhythmia (HR 1.24; 95% CI: 1.02–1.50; *P* = 0.032). Conversely, hepatocellular carcinoma was associated with a markedly lower risk (HR 0.45; 95% CI: 0.27–0.74; *P* = 0.002), suggesting a comparatively favorable safety profile for ICIs in this specific patient population (26). Furthermore, tumor progression appears to be associated with an elevated risk of arrhythmias; specifically, patients with advanced metastatic disease exhibit a significantly higher risk of ICI-associated arrhythmias (OR 3.48; 95% CI: 1.36–8.87; *P* = 0.009) (25).

#### 4.1.2 Pre-existing cardiovascular diseases

Patients with pre-existing cardiovascular diseases, including hypertension, coronary artery disease, and heart failure, possess impaired cardiac reserve and are consequently more susceptible to arrhythmias following ICI therapy. Indeed, a retrospective cohort study identified hypertension as an independent risk factor for ICI-associated arrhythmias (OR 1.75; 95% CI: 1.02–2.98; *P* = 0.04) (25). Li et al. compared patients with and without post-ICI arrhythmias, revealing that the former group had a significantly higher prevalence of baseline comorbidities, including heart failure (5.9% vs. 2.6%, *P* < 0.001), myocardial infarction (4.8% vs. 2.3%, *P* = 0.0002), peripheral vascular disease (15.6% vs. 12.3%, *P* = 0.0129), hypertension (50.8% vs. 45.1%, *P* = 0.0046), and renal disease (13.7% vs. 10.8%, *P* = 0.0221) (26).

#### 4.1.3 Lifestyle and inflammatory factors

Beyond pre-existing cardiovascular conditions, modifiable lifestyle factors and systemic inflammatory states further contribute to the risk of ICI-associated arrhythmias. For instance, smoking can alter the genomic landscape of lung cancer by recruiting T cells to the tumor microenvironment and upregulating PD-L1 expression, thereby enhancing ICI responsiveness (56). Consistently, clinical evidence has demonstrated a significant association between smoking and the development of ICI-associated arrhythmias (IRR 4.21; 95% CI: 1.29–13.76; *P* = 0.017) (24). Metabolic conditions, including obesity, have also been linked to enhanced ICI efficacy via the upregulation of PD-1 expression. However, an elevated body mass index remains a significant risk factor for ICI-associated arrhythmias (HR 1.05; 95% CI: 1.01–1.10; *P* = 0.033) (18). Systemic inflammation further plays a pivotal role in this setting. A prospective study confirmed that an elevated baseline neutrophil-to-lymphocyte ratio (NLR) was significantly associated with arrhythmic events (HR 1.74; 95% CI: 1.16–2.61; *P* = 0.007) (17). Furthermore, another study demonstrated that at the onset of arrhythmias, both NLR (20.7 ± 4.2 vs. 10.9 ± 8.3, *P* = 0.032) and C-reactive protein levels (109.9 ± 15.6 mg/L vs. 42.1 ± 10.1 mg/L, *P* = 0.010) increased significantly relative to baseline (36).

#### 4.1.4 Genetic predisposition

Human leukocyte antigen (HLA) alleles, such as HLA-B27, have been tentatively linked to arrhythmic risks via indirect pathways involving platelet function alteration. Specifically, HLA-B27 has been suggested to predispose patients to arrhythmias by impairing platelet function and promoting platelet adhesion to the vascular endothelium (57). Furthermore, specific HLA alleles can efficiently present cardiac autoantigens, such as troponin and myosin (58). Following ICI administration, this robust antigen presentation triggers activated T cells to cross-recognize and attack cardiomyocytes, thereby significantly elevating the risk of arrhythmias. It must be heavily emphasized that these assertions are strictly hypothesis-generating in nature; further extensive mechanistic studies and large-scale genetic mapping are mandatory before genetic screening can be considered an established pathogenetic mechanism for ICI-associated cardiotoxicity.

### 4.2 Treatment-related risk factors

#### 4.2.1 ICI classes

The risk of arrhythmias varies substantially across different ICI classes; however, consensus remains elusive due to conflicting findings. Multiple real-world studies utilizing the FAERS and European pharmacovigilance databases have indicated a significant association between arrhythmias and both PD-1 and PD-L1 inhibitors (39, 52, 55, 59). These investigations employed reporting odds ratios (RORs) to evaluate the strength of the association between specific agents and arrhythmic events. Notably, Balzano et al. provided robust evidence showing that PD-1 inhibitors (ROR 2.12; 95% CI: 1.68–2.68) and PD-L1 inhibitors (ROR 2.60; 95% CI: 2.02–3.35) were associated with significantly higher reporting rates for arrhythmias compared to CTLA-4 inhibitors (59). A potential explanation is that CTLA-4 is primarily expressed on T cells and operates during the priming phase of immune responses, whereas the PD-1/PD-L1 pathway is involved in the effector phase of immune regulation. Consequently, PD-1/PD-L1 inhibitors may sustain immune activation over a more extended period, thereby increasing the propensity for arrhythmias. Nevertheless, the FAERS and European pharmacovigilance databases are spontaneous reporting systems that rely on voluntary submissions from physicians, patients, and other healthcare professionals. This inherent limitation may introduce reporting bias, potentially resulting in fragmented, duplicated, or incomplete data. In contrast, two independent studies utilizing U.S. healthcare organization networks and commercial medical claims databases indicated a greater arrhythmogenic potential for CTLA-4 inhibitors. One of these studies identified a significantly increased risk of arrhythmias associated with CTLA-4 inhibitors within one year of treatment (HR 1.77; 95% CI: 1.34–2.34; *P* < 0.01) (26); the other demonstrated that the risk of atrial fibrillation associated with CTLA-4 inhibitors was 2.88-fold higher than that of PD-1 inhibitors (31). Furthermore, a meta-analysis encompassing 48 studies and 11,207 patients revealed a markedly higher incidence of arrhythmias in those treated with CTLA-4 inhibitors compared to PD-1/PD-L1 inhibitors (1.07% vs. 0.06%) (60). This disparity was attributed to immune dysregulation following CTLA-4 blockade, which promotes immune cell infiltration into the cardiac conduction system.

Contradictory findings also exist regarding the comparative risks of PD-1 and PD-L1 inhibitors. Balzano et al. reported that PD-L1 inhibitors were associated with higher arrhythmia reporting rates than PD-1 inhibitors (ROR 1.22; 95% CI: 1.06–1.41) (59). In a study of 5,744 patients with lung cancer, Pillai et al. found comparable incidences of overall irAEs between PD-1 and PD-L1 inhibitors, although cardiovascular events were not specifically assessed (61). Notably, Zhou et al. first proposed that PD-1 inhibitors may be more likely to induce CV-irAEs than PD-L1 inhibitors (29). A potential explanation is that, unlike PD-1 inhibitors, which block the interaction between PD-1 and both PD-L1 and PD-L2, PD-L1 inhibitors selectively inhibit the PD-1/PD-L1 interaction. This mechanistic difference confers a more potent immune-mediated effect on PD-1 inhibitors, thereby potentially increasing the risk of CV-irAEs. However, this study utilized composite cardiovascular events as the primary outcome, with arrhythmias representing only a small proportion (18.8%) of the total cases. Consequently, the specific arrhythmogenic effects of different ICI classes remain an area requiring further intensive investigation.

Among individual PD-1 inhibitors, pembrolizumab was associated with a higher arrhythmia reporting rate than its counterparts (ROR 1.26; 95% CI: 1.09–1.47); conversely, nivolumab exhibited the lowest arrhythmia reporting rate within this class (ROR 0.78; 95% CI: 0.68–0.91) (59). These differences may be attributed to structural variations between the two agents: pembrolizumab is a humanized monoclonal antibody comprising a murine antigen-binding region grafted onto a human framework, whereas nivolumab is a fully human monoclonal antibody entirely derived from human sequences (62). Studies have suggested that a higher degree of antibody humanization correlates with lower immunogenicity, which potentially translates into a reduced incidence of irAEs (63). Among individual PD-L1 inhibitors, avelumab exhibited the highest arrhythmia reporting rate (ROR 1.67; 95% CI: 1.08–2.58) (39). However, as the number of patients receiving avelumab in this cohort was relatively small, further large-scale studies are required to substantiate this finding.

#### 4.2.2 Drug dosage

Regarding drug dosage, Bertrand et al. conducted a meta-analysis demonstrating a dose-dependent relationship between CTLA-4 inhibitors and the risk of irAEs. Specifically, the incidence of irAEs was 61% when ipilimumab was administered at 3 mg/kg, increasing to 79% upon dose escalation to 10 mg/kg (64). However, cardiovascular events were not among the irAEs assessed in this study. Notably, subsequent research has found no such association between ICI dosage and CV-irAEs, with no significant differences observed in the occurrence of arrhythmias across varying dosages of PD-1, PD-L1, and CTLA-4 inhibitors (65, 66).

#### 4.2.3 Combination therapy

While ICI combination therapy has markedly enhanced antitumor efficacy and extended patient survival, it is also associated with a heightened risk of arrhythmias. A meta-analysis involving 25 studies and 20,244 patients demonstrated that combination therapy with PD-1 and CTLA-4 inhibitors significantly increased the risk of arrhythmias compared with PD-1 or CTLA-4 inhibitors monotherapy (67). Balzano et al. reported that the arrhythmia reporting rate for the combination of ipilimumab and nivolumab was significantly higher than that for ipilimumab (ROR 3.91; 95% CI: 3.01–5.07) or nivolumab monotherapy (ROR 2.07; 95% CI: 1.74–2.47) (59). Additionally, compared with chemotherapy alone, the combination of PD-1/PD-L1 inhibitors and chemotherapy was associated with a significantly increased risk of arrhythmias (OR 1.63; 95% CI: 1.07–2.46; *P* = 0.02), the risk that was particularly pronounced in PD-L1 inhibitor-chemotherapy regimens (OR 1.75; 95% CI: 1.09–2.80; *P* = 0.02) (68).

## 5 Pathophysiological mechanisms

The specific pathogenesis of ICI-associated arrhythmias remains incompletely elucidated. Conventionally, arrhythmias have been viewed as secondary complications of myocarditis. However, myocarditis cannot solely account for the increased burden of arrhythmias associated with ICI therapy; alternative pathophysiological mechanisms must be considered, particularly given that the clinical incidence of arrhythmias markedly exceeds that of myocarditis.

Emerging evidence demonstrates that T cells and macrophages activated by ICIs can specifically infiltrate the cardiac conduction system (sinoatrial node, atrioventricular node, and His-Purkinje system), thereby impairing the functional integrity of specialized conduction cells (69). Concurrently, these immune cells release a broad spectrum of proinflammatory cytokines, most notably interferon-γ, tumor necrosis factor-α, and interleukin-6, which exert potent proarrhythmic effects through multiple pathophysiological mechanisms (70). These direct effects are mediated through the intricate modulation of ion channels and key regulatory proteins in cardiomyocytes. Specifically, these cytokines prolong the action potential duration by inhibiting outward repolarizing currents (*I_to_*, *I_Ks_*, and *I_Kr_*) and enhancing inward depolarizing currents (*I_CaL_* and *I_Na_*). Moreover, they disrupt intracellular calcium homeostasis by interfering with calcium-handling proteins, which promotes spontaneous calcium release from the sarcoplasmic reticulum. This leads to intracellular calcium overload and increases the risk of early afterdepolarizations and delayed afterdepolarizations, thereby precipitating triggered activity. In addition, they downregulate connexin 43 between cardiomyocytes, leading to gap junction dysfunction and impaired impulse conduction. Regarding structural remodeling, proinflammatory cytokines activate myofibroblasts and drive extracellular matrix synthesis, inducing myocardial fibrosis that further compromises the velocity and uniformity of cardiac conduction. Indirect mechanisms encompass the inhibition of hepatic cytochrome P450 enzymatic activity, which significantly increases the bioavailability of co-administered drugs that prolong the corrected QT (QTc) interval. Additionally, ICIs can alter autonomic nervous system tone, resulting in excessive sympathetic activation that pathologically enhances sinoatrial node automaticity and shortens the refractory periods of the atria and ventricles. Collectively, these direct and indirect pathophysiological changes culminate in slowed and heterogeneous cardiac conduction. This not only establishes the substrate for conduction blocks but also facilitates the formation of reentrant circuits, which represent the fundamental mechanism underlying the development of reentrant arrhythmias.

Molecular mimicry also plays a crucial role in the pathogenesis of ICI-associated arrhythmias. Driven by the presence of shared antigenic epitopes between tumor cells and cardiomyocytes, such as troponin and myosin, T cells activated by ICIs can erroneously recognize and attack cardiomyocytes expressing these homologous antigens via immune cross-reactivity, thereby inducing arrhythmias (69). Furthermore, this systemic immune activation can trigger the generation of cardiomyocyte-specific autoantibodies, such as anti-myosin antibodies, which may further exacerbate cardiac electrical instability (71). Lee et al. found that approximately 60% of patients with atrial fibrillation harbor anti-myosin autoantibodies (72), which bind specifically to cardiac antigens and disrupt the physiological function of cardiomyocytes, ultimately resulting in electrophysiological disturbances and arrhythmias. Notably, the PD-1/PD-L1 pathway itself may be directly implicated in the development of arrhythmias, particularly atrial fibrillation. Liu et al. demonstrated that the expression of PD-1 on CD4^+^ T lymphocytes and PD-L1 on myeloid dendritic cells was significantly downregulated in patients with atrial fibrillation (73). This downregulation leads to the aberrant activation of CD4^+^ and CD8^+^ T lymphocytes, which in turn drives atrial electrical remodeling. In addition, ICIs can trigger the excessive production of reactive oxygen species (ROS), which induces oxidative stress and impairs mitochondrial function in cardiomyocytes, thereby promoting arrhythmogenesis (74).

ICIs may also compromise vascular endothelial function, leading to coronary microcirculatory dysfunction and aggravating myocardial ischemia, which collectively predispose the myocardium to arrhythmias. Moreover, arrhythmias can occur secondary to other irAEs, such as thyroid dysfunction and electrolyte disturbances triggered by ICIs (75) (Figure 1).

**Figure 1:**
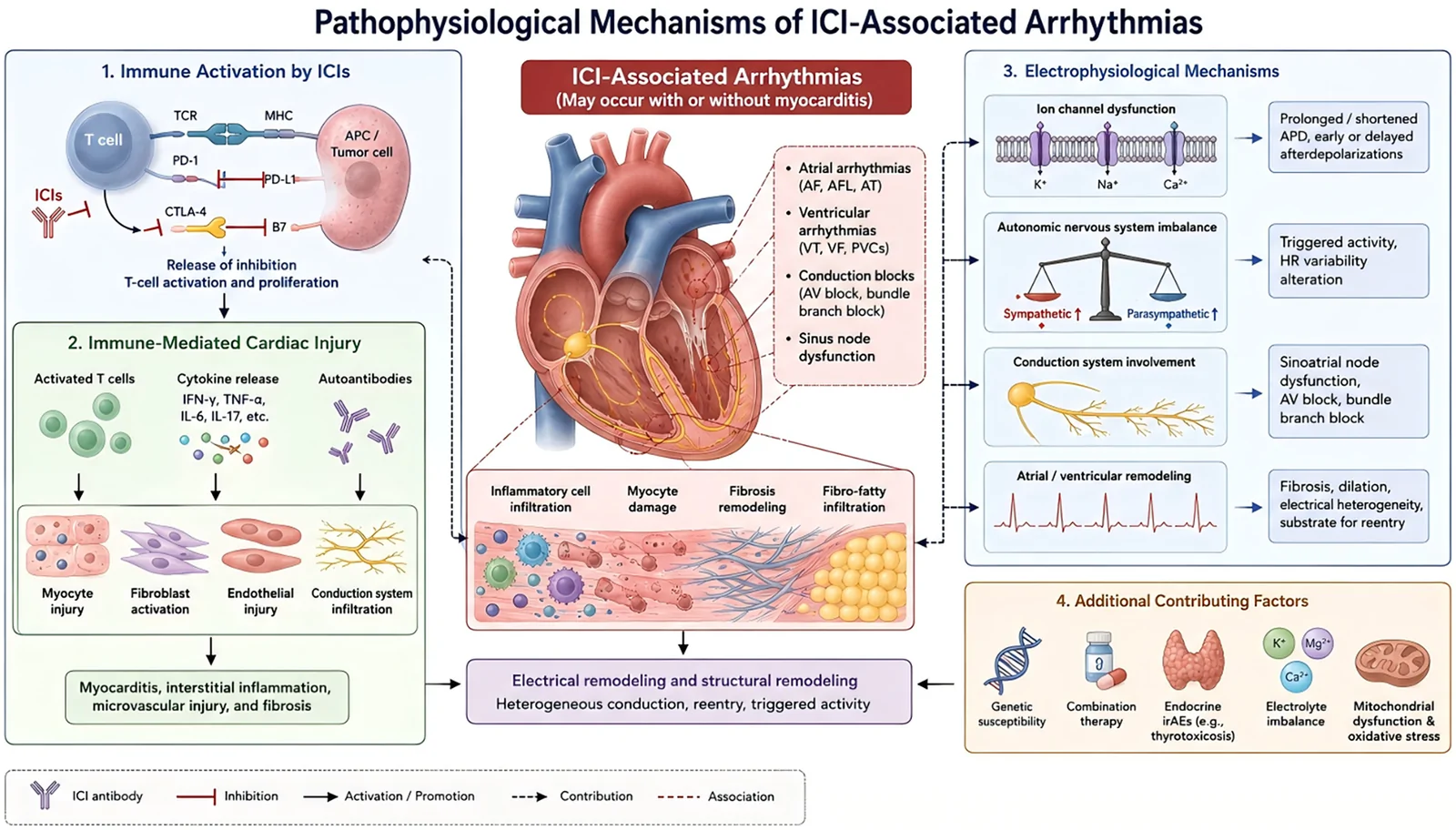
Proposed pathophysiological mechanisms of ICI-associated arrhythmias. ICIs block the PD-1/PD-L1 and CTLA-4 inhibitory pathways, thereby promoting T-cell activation, proliferation, and cytokine release. Activated immune cells, proinflammatory cytokines, and autoantibodies may induce cardiomyocyte injury, fibroblast activation, endothelial injury, and direct involvement of the cardiac conduction system. These immune-mediated effects, together with additional contributing factors—including genetic susceptibility, combination therapy, endocrine immune-related adverse events, electrolyte imbalance, mitochondrial dysfunction, and oxidative stress—may promote ion-channel dysfunction, autonomic imbalance, conduction-system injury, and atrial or ventricular remodeling. The resulting electrical and structural alterations may lead to abnormal automaticity, triggered activity, slowed or heterogeneous conduction, and re-entry, manifesting clinically as atrial arrhythmias, ventricular arrhythmias, conduction blocks, or sinus node dysfunction. ICI, immune checkpoint inhibitor; PD-1, programmed cell death protein 1; PD-L1, programmed cell death ligand 1; CTLA-4, cytotoxic T-lymphocyte-associated protein 4; TCR, T-cell receptor; MHC, major histocompatibility complex; APC, antigen-presenting cell; IFN-γ, interferon-γ; TNF-α, tumor necrosis factor-α; IL, interleukin; AF, atrial fibrillation; AFL, atrial flutter; AT, atrial tachycardia; VT, ventricular tachycardia; VF, ventricular fibrillation; PVCs, premature ventricular complexes; AV, atrioventricular; APD, action potential duration; HR, heart rate.

## 6 Clinical subtypes and ECG abnormalities

### 6.1 Atrial fibrillation

Atrial fibrillation represents the most prevalent arrhythmia associated with ICI therapy (52, 59). During atrial fibrillation episodes, cardiac pump function is significantly compromised, leading to hemodynamic instability that not only increases myocardial workload and precipitates complications such as heart failure but also promotes intra-atrial thrombus formation (76). This substantially elevates the risk of systemic thromboembolism, particularly ischemic stroke, posing a severe threat to patient survival. Balzano et al. analyzed 1,228 reported cases of ICI-associated atrial fibrillation, of which 248 (20.3%) were classified as severe and linked to fatal outcomes (59). Furthermore, a complex bidirectional relationship exists between atrial fibrillation and malignancy. A large prospective cohort study demonstrated that the cancer risk increased 3.5-fold within 3 months of an atrial fibrillation diagnosis; conversely, the risk of developing atrial fibrillation rose 4.6-fold within 3 months following a cancer diagnosis (77). Joseph et al. observed atrial fibrillation recurrence in patients with a history of paroxysmal atrial fibrillation following ICI initiation, highlighting the necessity for vigilant monitoring in this vulnerable subgroup (78). Perelman et al. evaluated 119 cancer patients with type 2 diabetes mellitus receiving ICI therapy and found that the use of sodium-glucose cotransporter 2 inhibitors was associated with a reduced incidence of atrial fibrillation (20). This finding offers novel insights into the prevention of ICI-associated arrhythmias and warrants further validation in broader clinical cohorts.

Importantly, atrial fibrillation occurring during ICI therapy should be interpreted as ICI-associated rather than as definitive evidence of a direct arrhythmogenic effect of ICIs, because it may arise secondary to factors such as ICI-related myocarditis, pericarditis, thyroid dysfunction, and electrolyte disturbances. Accordingly, a primary immune-mediated arrhythmic mechanism should be considered only after alternative precipitants have been carefully excluded.

### 6.2 Conduction blocks

ICI-associated conduction blocks may manifest as PR interval prolongation, bundle branch block, or atrioventricular block, potentially culminating in fatal cardiac arrest in severe cases (79). Among these, third-degree atrioventricular block is the most prevalent, while some patients may present with first- or second-degree atrioventricular block (80). In certain instances, multiple conduction blocks occur concurrently, reflecting diffuse involvement of the conduction system. For example, Xu et al. reported a case of hepatocellular carcinoma treated with ICIs where an ECG revealed new-onset first-degree atrioventricular block accompanied by right bundle branch block and left posterior fascicular block (81). ICI-associated conduction blocks can occur either in isolation or in conjunction with myocarditis. Power et al. reported that in a cohort of 147 patients with ICI-associated myocarditis, 11 (7.5%) developed second-degree atrioventricular block, while 25 (17.0%) progressed to third-degree atrioventricular block (82). In contrast, a study by Mir et al. involving 99 patients with ICI-associated cardiotoxicity observed that 12 (12%) presented with isolated conduction blocks (83). Furthermore, the occurrence of conduction blocks was significantly associated with increased cardiovascular mortality (80% vs. 16%, *P* = 0.003) (12).

### 6.3 Ventricular arrhythmias

Ventricular arrhythmias represent the most life-threatening manifestation of ICI-associated arrhythmias, frequently portending a dismal prognosis. These arrhythmias range from frequent premature ventricular contractions to ventricular tachycardia and ventricular fibrillation. Sabanci et al. reported a case of renal cell carcinoma in which the patient developed ventricular tachycardia shortly after initiation of ICI therapy. Despite no evidence of myocarditis on diagnostic workup, the patient's condition deteriorated rapidly, ultimately resulting in death (84). Similarly, in the context of ICI-associated myocarditis, the occurrence of ventricular tachycardia significantly escalates mortality risk (HR 6.82; 95% CI: 2.87–16.21; *P* < 0.001) (85).

### 6.4 Other ECG abnormalities

ECG serves as an indispensable tool for the clinical diagnosis and risk stratification of arrhythmias. Key ECG parameters, including the QTc interval, QT dispersion (QTd), and T-peak to T-end (Tp-e) interval, provide critical insights into ventricular repolarization dynamics. The QTc interval, encompassing the period from the onset of ventricular depolarization to the completion of repolarization, reflects global ventricular electrical activity. QTd, defined as the difference between the maximum and minimum QTc intervals across all leads, serves as a marker of the spatial heterogeneity of ventricular repolarization. Furthermore, the Tp-e interval reflects the transmural dispersion of repolarization. Prolongation of these parameters is closely linked to an increased susceptibility to malignant ventricular arrhythmias and sudden cardiac death. A cohort study following patients on ICI therapy for 6 months demonstrated that the QTc interval (430.75 ± 25.40 ms vs. 420.45 ± 20.60 ms, *P* = 0.013), QTd (50.20 ± 15.30 ms vs. 40.10 ± 10.55 ms, *P* = 0.001), and Tp-e interval (85.30 ± 12.40 ms vs. 80.21 ± 10.45 ms, *P* = 0.021) were all significantly prolonged relative to baseline (86). Consistent with these findings, Wu et al. reported that the QTc interval was significantly prolonged at the onset of arrhythmias compared with baseline (449.55 ± 37.17 ms vs. 432.79 ± 27.06 ms, *P* = 0.002) in patients treated with ICIs (28). Moreover, Pohl et al. observed a marked prolongation of QTd in patients receiving combined nivolumab and ipilimumab compared with baseline (50 ± 14 ms vs. 31 ± 14 ms, *P* < 0.0001), whereas this phenomenon was absent in those receiving nivolumab monotherapy (87).

## 7 Conventional and AI-enhanced monitoring

The clinical presentations of ICI-associated arrhythmias are predominantly non-specific; patients may present with palpitations, fatigue, dyspnea, dizziness, or syncope. Notably, a subset of patients remains asymptomatic, leading to an insidious onset that poses significant challenges to early detection. Although the incidence of these arrhythmias is substantial, it remains under-reported due to the underutilization of routine ECG monitoring or over-reliance on isolated 12-lead ECGs (88). Consequently, vigilant arrhythmic surveillance is paramount, particularly during the initial treatment phase when the risk of immune-related toxicities is highest. Current exploratory protocols suggest that patients initiating high-risk ICI regimens may benefit from baseline screening followed by serial ECG monitoring every 1–2 weeks during the critical initial 6 weeks of treatment (89). However, it must be noted that this approach does not currently represent a universal consensus or standardized standard of care across all international guidelines, reflecting the ongoing debate regarding the cost-benefit ratio of universal intensive surveillance. Although reported arrhythmic events cluster predominantly during the early treatment phase, the temporal spectrum of ICI-related cardiovascular toxicity is not restricted to the initial treatment cycles. In a recent case series, late-onset ICI-associated myocarditis was diagnosed after 6–20 months of ongoing ICI therapy, highlighting that myocarditis may occur beyond the early treatment period (90). However, evidence regarding the incidence and natural history of delayed isolated ICI-associated arrhythmias remains sparse. In addition to intensive vigilance during the early high-risk period, clinicians should maintain symptom-triggered and risk-adapted cardiovascular follow-up throughout treatment and after ICI cessation, particularly in patients with previous cardiovascular immune-related adverse events, combination ICI therapy, newly developed ECG abnormalities, or cardiac biomarker elevation. Nevertheless, routine long-term intensive ECG or wearable monitoring for all patients cannot currently be recommended in the absence of prospective evidence.

Beyond these conventional monitoring modalities, AI has emerged as a research frontier in precision cardio-oncology due to its inherent capability to analyze complex datasets and discern patterns imperceptible to the human eye. As the most widely accessible and standardized cardiac diagnostic tool, ECG—when integrated with AI—has revolutionized cardiovascular care for cancer patients (Table 3). For instance, Christopoulos et al. utilized an AI-ECG algorithm to assess the risk of atrial fibrillation in patients with chronic lymphocytic leukemia; their findings revealed that high AI-ECG scores were associated with a significantly increased risk of new-onset atrial fibrillation (HR 2.5; 95% CI: 1.6–3.9; *P* < 0.001) (91). Furthermore, in a cohort of 1,011 patients treated with anthracyclines, an AI model has been shown to predict cancer therapy-related cardiac dysfunction (CTRCD) from baseline ECG data (92), facilitating the identification of vulnerable patients even before treatment initiation. In the specific context of ICI-associated arrhythmias, AI-enhanced approaches leveraging machine learning, deep learning, and smart wearable devices offer transformative potential for early detection, risk stratification, and personalized monitoring. Heilbroner et al. developed an XGBoost-based machine learning model to predict cardiotoxicity in patients undergoing ICI therapy. By stratifying patients into low-risk and high-risk cohorts based on model outputs, they demonstrated a significant difference in the incidence of arrhythmias between the two groups (3.3% vs. 6.1%, *P* < 0.001) (34). Convolutional neural networks (CNNs), a specialized deep learning architecture, have become particularly transformative in the analysis of subtle ECG signal alterations due to their exceptional capability to capture spatial hierarchies in ECG data. Oikonomou et al. established an ECG-based CNN for cardiotoxicity risk stratification in patients receiving chemotherapy or ICI. Their findings indicated that a positive AI-ECG screening result was associated with a 3.4-fold higher incidence of CTRCD and a 13.5-fold higher incidence of left ventricular ejection fraction <40% (93). Building upon these findings, Ayoub et al. constructed a multimodal fusion AI model that exhibited robust performance in predicting CV-irAEs among ICI-treated patients, yielding an area under the curve of 0.72 (94). Notably, this multimodal approach outperformed standalone ECG, baseline electronic medical record data, and laboratory findings in predictive accuracy. Smart wearable devices facilitate the real-time, continuous acquisition of ECG data, enabling the precise characterization of dynamic alterations in cardiac electrical activity over prolonged intervals. By obviating the need for frequent outpatient ECG evaluations, these lightweight devices permit remote monitoring, which substantially mitigates healthcare-associated burdens and enhances patient adherence. This paradigm is especially advantageous for frail, mobility-impaired patients with malignancies, as it allows for the uninterrupted and comprehensive surveillance of cardiotoxicity throughout the continuum of antineoplastic therapy. Currently, the paramount clinical application of smart wearables within cardio-oncology resides in the continuous monitoring and proactive management of arrhythmias. Conventional ECG monitoring approaches are frequently inadequate for capturing paroxysmal or occult arrhythmias, which may subsequently progress to severe complications. Notably, smart wearable devices have proven effective in bridging this diagnostic gap. Studies have confirmed that ECG features derived from Lead I recordings of smartwatches demonstrate high concordance with those obtained from standard 12-lead ECG or Holter monitoring (95, 96). In a large-scale study involving 419,297 participants, Perez et al. reported that irregular pulse detection via smartwatches achieved a positive predictive value of 0.84 for diagnosing atrial fibrillation (97). Given the unique utility of AI-ECG in arrhythmia surveillance and the escalating clinical burden of ICI-associated arrhythmias, further integration of AI-enhanced approaches into cardio-oncology research is imperative. Pre-treatment risk stratification facilitated by AI algorithms will inform the development of targeted prevention and precision monitoring strategies, ultimately optimizing quality of life and long-term survival outcomes for cancer patients. However, the current evidence supporting AI for ICI-associated arrhythmias remains predominantly indirect. Among the studies included in this review, no AI model has yet been prospectively evaluated in a multicenter clinical implementation study using ICI-associated arrhythmia as the primary endpoint. AI-ECG algorithms, machine-learning risk scores, and wearable monitoring systems should therefore currently be regarded as investigational adjuncts rather than replacements for guideline-directed clinical assessment, conventional ECG monitoring, cardiac biomarkers, or cardiovascular imaging.

**Table 3:** Artificial intelligence studies relevant to cardiovascular risk prediction in oncology.

Authors/year	Population	AI model and outcome	Validation and principal limitations	Ref
Christopoulos et al. 2024	CLL, *n* = 754; additional BTK-inhibitor cohort	AI-ECG AF probability algorithm; incident AF	Retrospective application. AF-specific but not ICI-specific; BTK-inhibitor subgroup not statistically significant; no ICI-arrhythmia validation.	(91)
Yagi et al. 2024	Anthracycline-treated patients, *n* = 1,011	Baseline ECG-based AI model; CTRCD	Retrospective derivation and testing. Neither ICI-specific nor arrhythmia-specific.	(92)
Heilbroner et al. 2021	ICI-treated patients, *n* = 4,960	XGBoost using clinical and EHR variables; broad cardiac events, including arrhythmia and HF	Internal retrospective testing; AUC 0.65. ICI-specific but not arrhythmia-specific; broad endpoint, modest discrimination, and no prospective external validation.	(34)
Oikonomou et al. 2025	Anthracycline and/or trastuzumab, *n* = 1,550	AI-LVSD model applied to baseline ECG images; CTRCD and LVEF <40%	Retrospective single-system application. Neither ICI-specific nor arrhythmia-specific; no ICI-treated cohort.	(93)
Ayoub et al. 2025	ICI-treated patients with troponin testing, *n* = 2,258	Multimodal ECG and clinical-data model; myocarditis/MACE composite	Internal retrospective testing; AUC 0.72. ICI-specific but not arrhythmia-specific; selected cohort, composite endpoint, and no prospective external validation.	(94)

AF, atrial fibrillation; AI, artificial intelligence; AUC, area under the curve; BTK, Bruton tyrosine kinase; CLL, chronic lymphocytic leukemia; CTRCD, cancer therapy-related cardiac dysfunction; ECG, electrocardiography; EHR, electronic health record; HF, heart failure; ICI, immune checkpoint inhibitor; LVSD, left ventricular systolic dysfunction; LVEF, left ventricular ejection fraction; MACE, major adverse cardiovascular events.

Upon the detection of new-onset tachycardia or conduction blocks, the initial diagnostic workup should include the exclusion of non-cardiac etiologies, particularly electrolyte disturbances, thyroid dysfunction, and renal impairment. In this context, L'Orphelin et al. highlighted the significance of monitoring lipid profiles. Their study demonstrated that melanoma patients treated with ICIs developed significant dyslipidemia—a condition that precipitates inflammatory cascades, oxidative stress, and accelerated atherosclerosis, thereby predisposing patients to arrhythmias (98). Furthermore, comprehensive evaluation via cardiac biomarkers (e.g., cardiac troponin and B-type natriuretic peptide) and echocardiography is essential to screen for underlying cardiac pathologies (99). For patients with suspected concomitant myocarditis, cardiac magnetic resonance imaging or positron emission tomography is indicated; nonetheless, endomyocardial biopsy remains the diagnostic gold standard and should be judiciously considered if non-invasive findings are inconclusive (100). Additionally, coronary angiography is warranted for those presenting with suspected acute coronary syndrome.

Current guideline-supported practice remains centered on baseline cardiovascular risk assessment, conventional ECG and biomarker evaluation, risk-adapted imaging, and prompt symptom- or abnormality-triggered diagnostic workup. By contrast, fixed-interval intensive ECG surveillance, universal wearable monitoring, AI-derived risk scores, and automated remote alerts should currently be considered investigational strategies rather than established standards of care.

## 8 Management strategies

Following the confirmation of ICI-associated arrhythmias, immediate risk stratification is paramount. In accordance with guidelines from the American Society of Clinical Oncology and the Common Terminology Criteria for Adverse Events v5.0, these arrhythmias are stratified into four severity grades (101). Grade 1: Mild ECG abnormalities without symptoms; Grade 2: Mild diagnostic abnormalities accompanied by mild symptoms; Grade 3: Moderate diagnostic abnormalities or symptoms with mild exertion; Grade 4: Moderate to severe decompensation or life-threatening conditions (Table 4). Notably, given the inherent risk of cardiac dysfunction, further evaluation and intervention are indicated across all grades. For Grade 1 arrhythmias, ICI therapy may be continued under close clinical monitoring; conversely, prompt suspension of therapy is a critical step for patients with Grade ≥2 arrhythmias. Owing to the long half-life of ICIs, their pharmacologic effects often persist beyond treatment cessation; nonetheless, timely interruption remains pivotal to halting the progression of cardiac injury. While permanent discontinuation is recommended for Grade 3 or 4 arrhythmias, the timing and feasibility of ICI rechallenge in Grade 2 arrhythmias remain controversial. In such instances, individualized decisions should be made by a cardio-oncology multidisciplinary team (MDT) comprising cardiologists, oncologists, and other relevant specialists, following a comprehensive assessment of oncologic prognosis, cardiac recovery, and potential risks (99).

**Table 4:** Graded management for ICI-associated arrhythmias.

Arrhythmia subtype	Estimated incidence	CTCAE v5.0 toxicity grade	Recommended management and ICI dosing decision
Atrial fibrillation	3.0%–6.5%	Grade 1: Asymptomatic	Continue ICI therapy under close monitoring.
		Grade 2: Symptomatic; manageable with non-invasive intervention	Interruption of ICI therapy. Rechallenge after MDT assessment.
		Grade 3/4: Hemodynamic instability; life-threatening decompensation	Permanent discontinuation of ICI therapy. Cardio-oncology admission.
Conduction blocks	1.0%–3.5%	Grade 1: Asymptomatic PR interval prolongation	Continue ICI therapy under close monitoring.
		Grade 2: Intermittent second-degree AVB; mild symptoms	Interruption of ICI therapy. Immediate continuous telemetry monitoring.
		Grade 3/4: High-grade AVB; syncope; cardiac arrest	Permanent discontinuation of ICI therapy. Urgent temporary pacemaker placement. High-dose corticosteroid.
Ventricular arrhythmias	1.0%–4.5%	Grade 2: Frequent, symptomatic premature ventricular contractions	Interruption of ICI therapy.
		Grade 3/4: Ventricular tachycardia or ventricular fibrillation	Permanent discontinuation of ICI therapy. Emergency defibrillation/cardioversion. High-dose corticosteroid.

ICI, immune checkpoint inhibitor; AVB, atrioventricular block; MDT, multidisciplinary team.

The management of arrhythmias involves a dual strategy targeting both the underlying etiology and the specific arrhythmic manifestation (Figure 2): (i) Management of precipitating factors and underlying etiologies: Correction of electrolyte disturbances and thyroid dysfunction is essential. Additionally, for suspected or confirmed concomitant myocarditis, high-dose corticosteroid therapy must be initiated without delay. In patients refractory to corticosteroids or those exhibiting clinical deterioration, prompt escalation to additional immunosuppressive agents is warranted (6). Notably, prophylactic corticosteroid use is generally discouraged, as it may compromise the antitumor efficacy of ICIs and lacks evidence for preventing cardiotoxicity. (ii) Management of specific arrhythmic manifestations: Conversely, when arrhythmias present as isolated clinical entities completely devoid of subclinical or overt myocarditis, the therapeutic paradigm shifts predominantly toward standard cardiovascular protocols. The primary goals are symptom control, hemodynamic stabilization, and the prevention of sudden cardiac death. Throughout this process, the potential interactions between cancer status and pharmacotherapeutic agents (antiarrhythmics and anticoagulants) must be meticulously evaluated (102). For atrial fibrillation, management strategies encompass anticoagulation, ventricular rate control, and cardioversion, with the latter necessitating the exclusion of left atrial appendage thrombi. Nonetheless, clinicians must recognize that this constitutes general atrial fibrillation care rather than an ICI-specific therapeutic discovery. However, applying these general antiarrhythmic strategies within the oncological cohort requires specialized cardio-oncological tailoring. The CHA_2_DS_2_-VASc score is a pivotal tool for assessing thromboembolic risk in atrial fibrillation. However, the scoring system may underestimate this risk in ICI-associated atrial fibrillation, as it fails to account for the hypercoagulable state induced by malignancy. Pastori et al. demonstrated that the predictive value of the CHA_2_DS_2_-VASc score for thromboembolism is lower in cancer patients than in non-cancer patients (103). Consequently, the European Society of Cardiology guidelines on cardio-oncology recommend anticoagulation for cancer patients with atrial fibrillation who have a CHA_2_DS_2_-VASc score ≥1 for men or ≥2 for women (104). It is critical to note that cancer patients often exhibit a significantly heightened bleeding risk during anticoagulation therapy due to comorbid anemia or thrombocytopenia (105). In this cohort, direct oral anticoagulants have demonstrated superior safety and efficacy compared to warfarin, associated with lower incidences of both bleeding and thromboembolic events (106). Given the dual burden of high thromboembolic and hemorrhagic risks in cancer patients, the restoration and maintenance of sinus rhythm are particularly crucial for ICI-associated atrial fibrillation. Nevertheless, studies have shown that the use of antiarrhythmic drugs (AADs) in cancer patients may pose unique safety challenges (107). For instance, amiodarone can prolong the QTc interval, and ICIs may further exacerbate this risk by increasing the bioavailability of amiodarone. Therefore, AAD therapy in this setting requires stringent monitoring; treatment should be interrupted upon the occurrence of significant QTc prolongation and cautiously reinitiated at reduced dosages only after the interval has returned to baseline. Electrical cardioversion should be considered for patients with atrial fibrillation presenting with hemodynamic instability. Clinical evidence regarding catheter ablation (CA) in atrial fibrillation patients with cancer remains limited. Farmakis et al. suggest that CA may be a viable option for cancer patients who are refractory to or have contraindications to AADs (108). However, CA in this cohort necessitates multifaceted considerations, including life expectancy and potential clinical benefit. A meta-analysis evaluating the safety of CA in patients with atrial fibrillation and cancer demonstrated a significantly higher risk of post-procedural bleeding than in non-cancer patients (109). Accordingly, decisions regarding CA should be made by a cardio-oncology MDT, accounting for the cancer status and the potential risk of complications. Life-threatening ventricular tachycardia and ventricular fibrillation necessitate immediate electrical cardioversion or defibrillation. For high-grade atrioventricular block, urgent temporary pacemaker implantation is required. Given that conduction disturbances may be reversible, permanent pacemaker implantation should be reserved solely for patients with persistent conduction system impairment or evidence of irreversible fibrosis (88).

**Figure 2:**
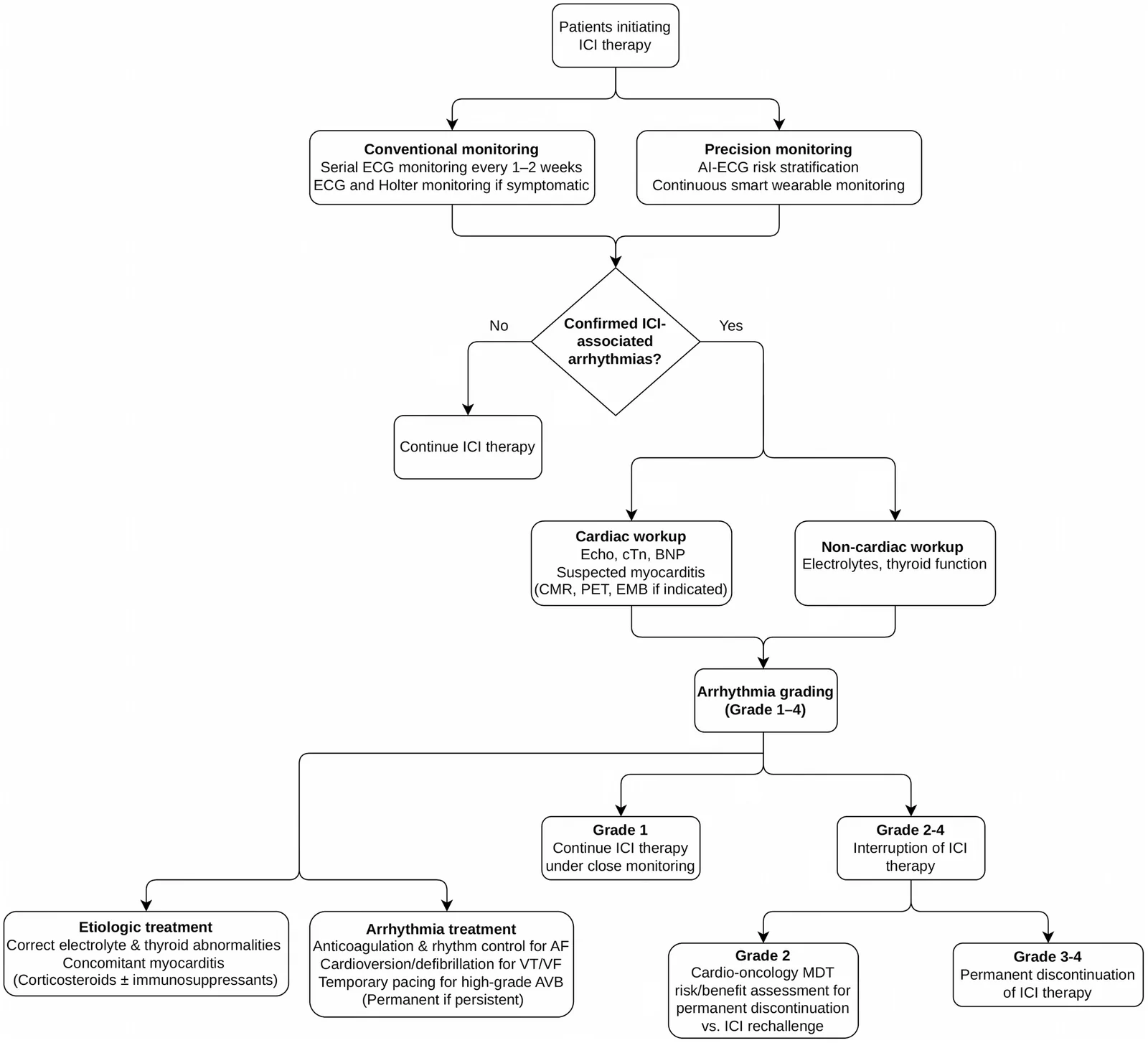
Flowchart for monitoring and management of ICI-associated arrhythmias. This flowchart illustrates a standardized clinical pathway for patients initiating ICI therapy, integrating conventional ECG monitoring with AI-enhanced precision monitoring. For patients with confirmed ICI-associated arrhythmias, it delineates a stepwise diagnostic workup (cardiac and non-cardiac evaluation) and dual-track therapeutic strategies covering etiological treatment and arrhythmia-specific treatment. Furthermore, it outlines the decision-making process for ICI interruption, permanent discontinuation, or rechallenge, underpinned by a severity-based graded management framework. ICI, immune checkpoint inhibitor; ECG, electrocardiography; AI, artificial intelligence; cTn, cardiac troponin; BNP, B-type natriuretic peptide; Echo, echocardiography; CMR, cardiac magnetic resonance; PET, positron emission tomography; EMB, endomyocardial biopsy; AF, atrial fibrillation; VT, ventricular tachycardia; VF, ventricular fibrillation; AVB, atrioventricular block; MDT, multidisciplinary team.

## 9 Limitations and future directions

While this review provides a comprehensive synthesis of the epidemiology, risk factors, and underlying pathophysiological mechanisms of ICI-associated arrhythmias, several critical limitations inherent to the current literature must be acknowledged. First, a profound limitation stems from the extreme methodological heterogeneity among the compiled clinical studies. The majority of available data relies on retrospective analyses or spontaneous pharmacovigilance reporting systems (e.g., FAERS), which are inherently vulnerable to under-reporting, diagnostic misclassification, and an inability to definitively establish causality. Conversely, prospective studies employing rigorous serial ECG monitoring remain limited in scale, complicating the accurate delineation of the true universal incidence of isolated arrhythmias. Second, separating direct ICI-mediated cardiotoxicity from secondary electrical manifestations triggered by overlapping irAEs—such as subclinical myocarditis or thyroid dysfunction—remains an ongoing diagnostic challenge in real-world cohorts. An additional limitation arises from the narrative design of this review. Although a structured literature search was performed, the review did not use a registered systematic-review protocol or formal risk-of-bias and certainty-of-evidence assessment. Therefore, selection bias and subjective interpretation of heterogeneous evidence remain possible.

To overcome these barriers, future clinical research must prioritize several strategic directions. First, standardized multi-center prospective registries should be established to launch large-scale, prospective multi-center cohorts utilizing standardized, intensive cardiac monitoring protocols to accurately capture paroxysmal or occult arrhythmic phenotypes. Second, deep characterization of biomarkers and multi-omics analysis should be performed, whereby future studies integrate serial high-sensitivity cardiac troponins and advanced multi-omic profiling (including HLA mapping and autoantibody panels) to discern specific patient populations with high genetic susceptibility. Third, further efforts are required for the validation of ICI-specific AI algorithms; while AI-enhanced monitoring and smart wearables represent a highly promising exploratory frontier, future engineering must focus on training CNNs on ICI-specific datasets to move beyond proof-of-concept models and validate near-ready precision screening solutions. Finally, targeted mechanistic bench-to-bedside research is essential, and translational bench studies should focus on establishing targeted animal models to isolate the precise cell-signaling pathways and cytokine-mediated ion channel shifts that drive isolated electrical instability in the absence of histopathological myocarditis.

## 10 Conclusions

As a landmark advancement in oncology, ICIs have demonstrated transformative efficacy across diverse malignancies, delivering substantial benefits for cancer control and patient survival. However, immune-related cardiotoxicity presents a formidable challenge to clinical outcomes, with arrhythmias garnering significant attention due to their high prevalence and potentially fatal nature. Characterized by early onset, varied clinical manifestations, and a lack of pathognomonic symptoms, ICI-associated arrhythmias pose a major hurdle to early detection and precision intervention. An in-depth understanding of the pathophysiological mechanisms and risk factors, coupled with comprehensive management strategies, is essential to balance oncological benefits with cardiovascular safety. By integrating these critical clinical insights with AI-enhanced precision monitoring, clinicians can maintain effective cancer control while maximizing cardiovascular protection, ultimately achieving high-quality, long-term survivorship.
